# Synergistic Effects of Magnetic Nanomaterials on Post-Digestate for Biogas Production

**DOI:** 10.3390/molecules26216434

**Published:** 2021-10-25

**Authors:** Emmanuel Kweinor Tetteh, Gloria Amo-Duodu, Sudesh Rathilal

**Affiliations:** Green Engineering and Sustainability Research Group, Department of Chemical Engineering, Faculty of Engineering and The Built Environment, Durban University of Technology, Durban 4001, South Africa; gamoduodu04@gmail.com (G.A.-D.); rathilals@dut.ac.za (S.R.)

**Keywords:** anaerobic digestion, biogas, digestate, magnetite, renewable energy, wastewater

## Abstract

Digestate is characterized by high water content, and in the water and wastewater treatment settings, necessitates both large storage capacities and a high cost of disposal. By seeding digestate with four magnetic nanoparticles (MNPs), this study aimed to recover biogas and boost its methane potential anaerobically. This was carried out via biochemical methane potential (BMP) tests with five 1 L bioreactors, with a working volume of 80% and 20% head space. These were operated under anaerobic conditions at a temperature 40 °C for a 30 d incubation period. The SEM/EDX results revealed that the morphological surface area of the digestate with the MNPs increased as compared to its raw state. Comparatively, the degree of degradation of the bioreactors with MNPs resulted in over 75% decontamination (COD, color, and turbidity) as compared to the control system result of 60% without MNPs. The highest biogas production (400 mL/day) and methane yield (100% CH_4_) was attained with 2 g of Fe_2_O_4_-TiO_2_ MNPs as compared to the control biogas production (350 mL/day) and methane yield (65% CH_4_). Economically, the highest energy balance achieved was estimated as 320.49 ZAR/kWh, or 22.89 USD/kWh in annual energy savings for this same system. These findings demonstrate that digestate seeded with MNPs has great potential to improve decontamination efficiency, biogas production and circular economy in wastewater management.

## 1. Introduction

Bioenergy production has been seen as one of the most environmentally friendly solutions available for the degradation of chemically complex digestates [1]. These include wastewater treatment plant sludge, paper mill sludge, organic fraction of municipal solid waste, industrial wastewater and waste streams from the food and pharmaceutical industries, which can undergo microbial metabolic pathways via anaerobic digestion (AD) to produce biogas [2]. In addition, digestate is readily available and exceptionally rich in macro- and micronutrients, propelling its usability for agricultural applications such as NPK fertilizer. Also, ammonia and free phosphorus, which have been freed from their biologically bound states in feedstock, have the potential to be recycled back into the food chain for agricultural farming. However, AD has many reported setbacks [1,2,3], which warrants trace-element involvement in order to propel its complex reactions and mechanisms in bioenergy production.

Recent complex sludge production from primary, secondary, and tertiary treatment in wastewater treatment plants (WWTPs) has resulted in pressing concerns, as its treatment cost accounts for 20–55% of the total operating costs of WWTPs. This necessitates the possibility of considering an abatement technology for sewage sludge treatment given such sensitive factors as the end product, energy generation, the environment and human health impacts [4,5]. Sewage waste is becoming a major concern in South Africa (SA), since the volume of solid waste produced is escalating daily. This is attributed to rapid population growth and industrialization. In this case, the capability of AD facilities for processing municipal waste is being overused to reduce waste before it is disposed of in landfills [6,7]. However, due to legal and budgetary constraints, landfill use is dwindling as a conventional approach whereas dumpsites remain limited. In response to the substantial risks involved with municipal solid waste management for final disposal, municipalities are seeking a better option to mitigate these pressing concerns. Of note, several studies on consistent AD operation in treating the organic components of sewage sludge are also being undertaken [6,8].

Subsequently, using sludge as a bioenergy resource is reported to be eco-friendly, with a high caloric value ranging from 21–23.5 MJ/m^3^, which will help ease the over dependency on fossil fuels to generate bioenergy [3]. In this vein, biogas produced from municipal waste can be used for electricity, fuel for vehicles and heat for cooking, and can therefore offset the limitations that fossil fuels pose [1,3]. Generally, production of biogas via AD involves four processes: (i) hydrolysis of complex organic compounds to manageable soluble compounds; (ii) acidogenesis, which converts the soluble compound to volatile fatty acids; (iii) the acetogenesis stage, where higher organic acids and alcohol from the preceding stage are converted to acetic acids, CO_2_ and H_2_; and finally (iv) the methanogenesis phase [3,6,7].

To develop AD technology and meet current energy and environmental concerns, it is critical to research wastewater-based biogas production as an alternative option. The use of nanotechnology in the wastewater settings, especially the magnetisation separation coupled with AD process knowledge is still limited. Therefore, this study aimed to employ magnetic nanomaterials (MNPs) as a biostimulant in the AD process for enrichment of the organic fraction of municipal WWTP digestate into methane-rich biogas. The morphological and elemental analysis of the digestate is highlighted. In addition, an existing kinetic model is used to establish the degree of degradation and the biogas production.

## 2. Results and Discussions

### 2.1. Digestate Morphological Properties

**Figure 1:**
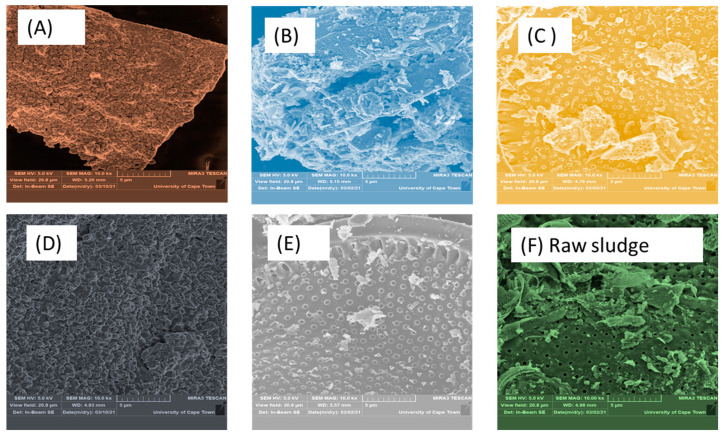
SEM images for post-sludge bioreactors A–D charged with 2 g of (**A**) Fe_2_O_4_-TiO_2_; (**B**) Cu-Fe_2_O_4_; (**C**) Fe_3_O_4_; and (**D**) Chitosan-Fe_2_O_4_/TiO_2_ MNPs, whereas bioreactor E is a control: (**E**) no MNPs and (**F**) raw sludge.

SEM/EDX was used to investigate the compatibility and morphology of the digestate before and after 30 days of incubation. The interfacial adhesion and dispersion of the MNPs in the digestate is presented in Figure 1. At a micrograph scale of 5 µm, the digestate surface profile was taken at a high magnification of 10k× with a width view size of 20.8 µm. The raw sludge (Figure 1F) shows a porous structure with an irregular shape, revealing the potential of active microbes for biodegradation. However, after 30 days of incubation, the particle surface with Fe_2_O_4_-TiO_2_ (Figure 1A) was found to be much smoother than the original digestate (Figure 1F). The micrograph indicates that the digestate with MNPs (Figure 1A–D) consisted more of cluster cells, while that without (Figure 1B,C) consisted more of filamentous clusters. Figure 1A,D also reveal that the micrograph images include flower-like particles, with many apertures distributed along the surface. This suggest the digestate, which consisted of Fe_2_O_4_-TiO_2_ and Chitosan- Fe_2_O_4_/TiO_2_ in bioreactors A and D, may have increased the contact surface (Figure 1A,D), reactivity and degradation efficiency in reducing contaminants as well as increasing biogas production [6,8].

**Figure 2:**
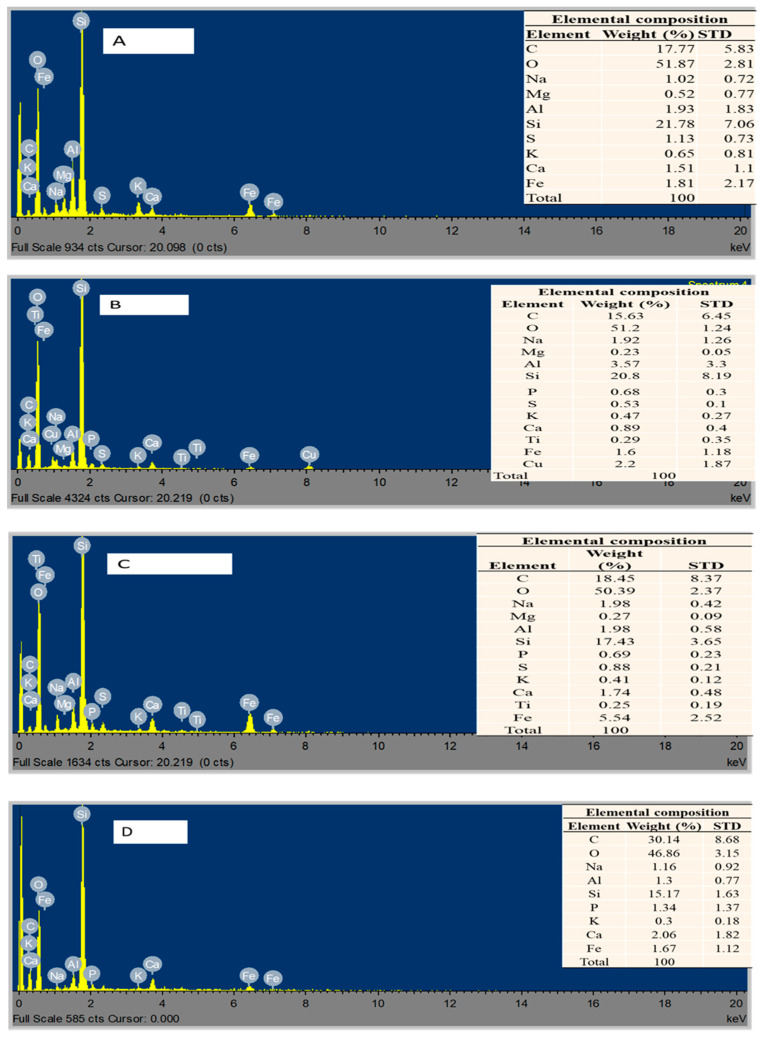

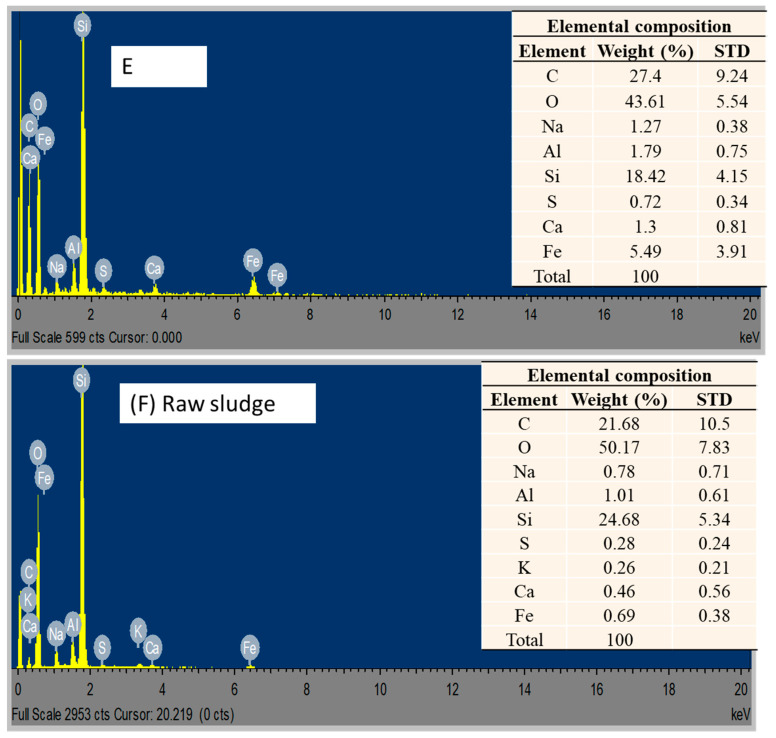
EDX images for post-digestate of bioreactors A–D charged with 2 g of (**A**) Fe_2_O_4_-TiO_2_; (**B**) Cu-Fe_2_O_4_; (**C**) Fe_3_O_4_; and (**D**) Chitosan-Fe_2_O_4_/TiO_2_ MNPs, whereas bioreactor E is a control: (**E**) no MNPs and (**F**) raw sludge.

The electron micrographs showing the spatial distribution of different elemental composition in the sludges are illustrated in Figure 2. Quantitatively, the most predominant elemental distribution on the surface of the digestate was found to be Al, Si, Na and Fe (Figure 2). These elements, aside from the carbonate (C, O), influenced the adsorption ability of the contaminants. Substrates rich in Al or Fe potentially stimulate inter-species reactions that oxidize complex metals (S or P) and remove them via precipitation [6,8]. SEM/EDX (Figure 1) revealed that the mineralisation of the digestate had interactive features which increased the methanogenic pathway of the organics. The EDX (Figure 2), showed that the metabolic pathways were very sensitive to the microbial community, as there was a reduction in their concentration level. The availability of the nutrient-rich trace elements also increased methanogenic activity. The presence of MNPs amplified the strong electrostatic contact between the core NPs and the pollutants present in the wastewater, as previously observed. Therefore, subsequent study also investigated the role of the MNPs in the removal of specified contaminants from the wastewater.

### 2.2. Decontamination of the Wastewater

In this study, AD was investigated as a medium to degrade high-strength organics and generate biogas with rich methane potential, as well as to decontaminate pollutants to meet the discharge limits of the South African Department of Environmental Affairs [9,10]. This is important due to many parameters in wastewater treatment being identified as posing threats to human health and aquatic life when released in large quantities into the environment [10,11]. Table 1 presents the water quality parameters including COD, colour, and turbidity which were considered in this study to ascertain whether they are aligned with their respective discharge limits [10]. It was found that over 70% of the pollutants were removed by the bioreactors with the MNP additives as compared to that of the control system. Among these bioreactors (A–E), bioreactor A was found to be more dominant in terms of its treatment performance as compared to the others. This affirms other studies where the presence of Fe_2_O_4_-TiO_2_ released trivalent ions as electron donors which instigated the agglomeration and neutralisation of pollutants for their removal [12,13,14,15,16].

### 2.3. Digestate Degradation and Biogas Production

**Figure 3:**
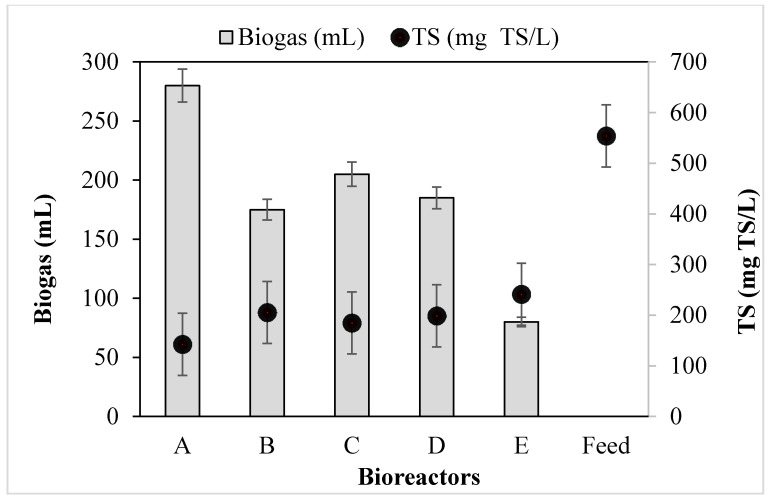
Biogas yield and TS degradation of bioreactors A–D charged with 2 g of (**A**) Fe_2_O_4_-TiO_2_; (**B**) Cu-Fe_2_O_4_; (**C**) Fe_3_O_4_; and (**D**) Chitosan-Fe_2_O_4_/TiO_2_ MNPs, whereas bioreactor E is a control: (**E**) no MNPs.

Biogas is constituted by methane, carbon dioxide and other trace gases as the prime component of AD of waste or wastewater along with high-strength organics [1,3]. To improve AD biogas output, the potential biochemical methane system was dosed with different MNPs and their influence was monitored for 30 days. Figure 3 shows the amount of TS compared with the biogas produced by bioreactors A–D charged with 2 g of Fe_2_O_4_-TiO_2;_ Cu-Fe_2_O_4_; Fe_3_O_4_ and Chitosan-Fe_2_O_4_/TiO_2_ MNPs, respectively, whereas bioreactor E had no MNP additives. The considerable TS outputs achieved were as follows: A (142.55 mg TS/L) < C (184.73 mg TS/L) < D (198.66 mg TS/L) < B (205.49 mg TS/L) < E (241.31 mg TS/L).

Comparatively, bioreactor A was found with the lowest digestate at 142.55 mgTS/L, corresponding to the highest biogas of 400 mL/d, whereas bioreactor E, being the control system, was found to be the lowest with biogas of 130 mL/day and biomass of 241.31 mg TS/L. Lower yield of the digestate was observed among the bioreactors charged with the MNPs, suggesting that there was significant microbial activity which resulted in the degradation of the organics during biogas production [2,17,18,19,20]. Conversely, for bioreactor E (control), the difference in the digestate compared to the other reactors was enormous, such that less microbial activity occurred [2,18].

Furthermore, the digestate can be used as a as a by-product for fertilizer or soil amendment, depending on the application. This is associated with the quality of the feedstock and the degree of degradation efficiency in the process, as well as the extent to which the post-treatment process was carried out. Significantly, sludge treatment contributes greatly to the total cost of wastewater treatment plants [4,5]. It was observed that the addition of MNPs to the bioreactors were easily separated from the sludge by an external magnet. This suggests that the bioreactors with MNP additives inducted the sludge with its super-magnetic properties, which strengthened its degradability [19,21]. Therefore, the low sludge generated (Figure 3) suggests that biostimulation of wastewater systems with magnetic separation processes can help mitigate the costs of treatment and landfill complexity.

**Figure 4:**
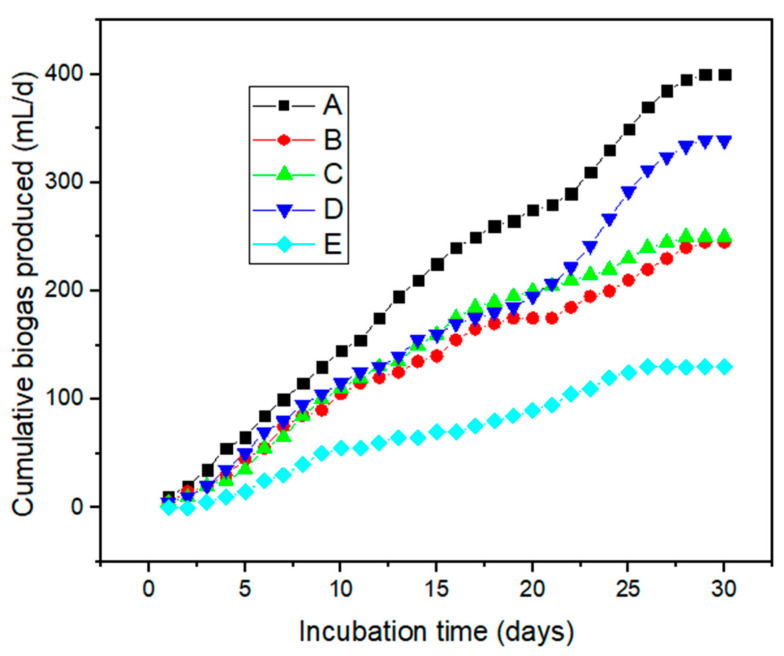
Cumulative biogas yield for 30 days of bioreactors A–D charged with 2 g of (**A**) Fe_2_O_4_-TiO_2_; (**B**) Cu-Fe_2_O_4_; (**C**) Fe_3_O_4_; and (**D**) Chitosan-Fe_2_O_4_/TiO_2_ MNPs, whereas bioreactor E is a control: (**E**) no MNPs.

Figure 4 presents the cumulative biogas production yield collected over the incubation period of 30 days. Slow or low biogas production was observed during the first week, accompanied by rapid production from the 5th to the 25th day, followed by steady production until the last week when the system was shut down. The slow production observed is in agreement with other reported works which suggest the microbes need to get acclimatised before they begin production [4,5,12]. The overall biogas production was enhanced by the MNP additives to the bioreactors as compared to the control system without any MNP additives. This resulted in an increase in bioreactor production efficiency as follows: A (400 mL/day) > D (339 mL/day) > C (250 mL/day) > B (245 mL/day) > E (130 mL/day). Significantly, the bioreactor charged with Fe_2_O_4_-TiO_2_ MNPs positively impacted biogas production (*p* < 0.05) [18]. MNP additives released metal ions into the substrate as nourishment, which stimulated microbial activity via long-term exposure [19]. In addition, the high surface area of the MNPs facilitated agglomeration, which had a great potential impact on the enzymatic activities of the methanogenesis microbes which increased the biogas production [18,19].

### 2.4. Biomethane Potential

**Figure 5:**
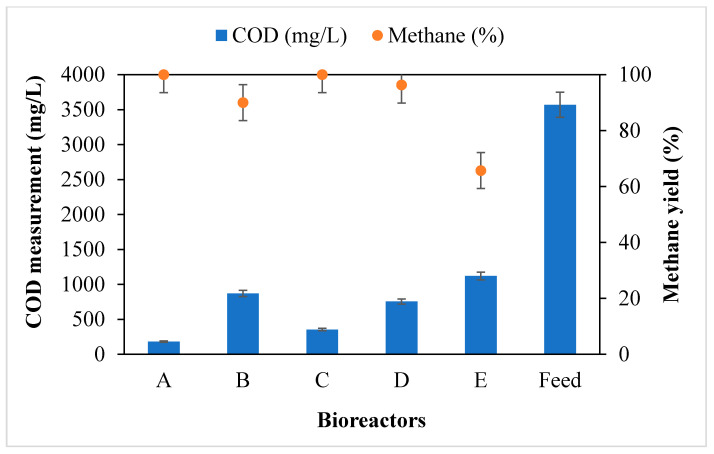
COD measurement and methane yield of bioreactors A–D charged with 2 g of (**A**) Fe_2_O_4_-TiO_2_; (**B**) Cu-Fe_2_O_4_; (**C**) Fe_3_O_4_; and (**D**) Chitosan-Fe_2_O_4_/TiO_2_ MNPs, whereas bioreactor E is a control: (**E**) no MNPs.

Figure 5 shows the biogas composition as characterised after 30 days of incubation. Here, the direct interspecies electron transfer by the MNPs played an important role in facilitating methanogenesis activity during the digestion period [18,20]. This positively resulted in over 75% degradation of the initial COD of 3570 ± 79 mg COD/L, which resulted in an increase in the methane yield (Figure 5). This result affirms other reported works in that the release of the electron carriers by the MNPs can regenerate into H_2_, which serves as an electron donor and combines with CO_2_ to produce CH_4_ [1,2,18,19,21]; this was observed in bioreactors A–D, which had an increase in methane yield. Additionally, the high surface area of the Fe_2_O_4_-TiO_2_ MNPs had a positive effect on the AD process, which resulted in both biogas enhancement and higher methane yield [18,22]. Evidently (Figure 5), a reduction in COD increased the productivity of the volatile fatty acids, which were then converted into the CH_4_ potential by the methanogens as recorded by each bioreactor: A (100% CH_4_); B (90% CH_4_); C (100% CH_4_); D (100% CH_4_); and E (65% CH_4_). Similar results were reported by other researchers, as MNPs influence biomethane potential [1,18,22,23]. This present study was no different, in that the addition of the MNPs had a significant impact on COD reduction and methane yield.

### 2.5. Technoeconomic Analysis of the Potential Estimated Energy

To ascertain the effectiveness of this technology and its economic feasibility, the cost of the energy produced was estimated based on the methane yield. Theoretically, the biogas consists of about 60–65% CH_4_ and 35–40% CO_2_, and 80% of the energy produced is converted into electricity [24,25]. Herein, the cost of energy was estimated based on the volume of biogas produced by the bioreactors: A (400 mL/day) > D (339 mL/day) > C (250 mL/day) > B (245 mL/day) > E (130 mL/day), with the results obtained presented in Table 2. To compare the energy economy of each bioreactor to that of [23] study, an assumed 80% methane potential (Figure 5) observed via the BMP test composition was used. This was based on 0.3 L of sludge, which was used to inoculate the degradation of the wastewater via methanogenesis to produce the biogas, as well as subsequent characterisation of the methane composition. The calorific values of the substrate as well as that of the digestate were also considered for balancing the energy during the anaerobic digestion process [18,26]. The estimated calorific energy required $\left(E_{H}\right)$ by the waterbath to maintain the digester temperature is given by (1):

(1) $$E_{H}=Q\times C_{p}\times p\;\left(T_{i}-T_{0}\right)$$

where Q = substrate flowrate (m^3^/d), $C_{p}$= specific heat of feed (kJ/kg °C), $T_{i}\;$= the digester temperature and $T_{0}\;$= the substrate temperature from its stock [23].

The daily energy production by each bioreactor $E_{A}\;$(kJ/d) corresponding to that of the methane contained in the produced biogas is given by (2). In addition, the net energy production $E_{P}\;$(kJ/d) is the difference between the produced energy and the energy consumed by the process (3).

(2) $$E_{A}=\left(Mp\right)\times \left(L.H.V\;of\;methane\right)$$

(3) $$E_{P}=E_{A}-E_{H}$$

where *Mp* = daily methane production rate (m^3^ CH_4_/d) and *L.H.V* = lower heating value of 35.8 KJ/m^3^ CH_4_ [24].

The main purpose of observing the energy balance in this experiment was to examine the economics of the energy potential of wastewater in terms of sustainable circular economy. Among the bioreactors A–E (Table 2), bioreactor A, charged with 2 g of Fe_2_O_4_-TiO_2_, was found to be the most economically viable system with an estimated net energy profit of 320.49 ZAR/kWh, or 22.89 USD/kWh. Evidently, as observed in Table 2, all the bioreactors charged with the MNPs were found to be more economical than the control system which had no MNPs. This validates the positive role the MNPs charged to the bioreactors played in enhancing biogas production (Figure 4) as well as methanation efficiency (Figure 5). This proves that the use of MNPs to enhance biogas yield will be cost effective and large-scale production will be economically feasible [23,24].

## 3. Materials and Methods

### 3.1. Chemicals and Feedstock Collection

#### 3.1.1. Synthesis and Characterisation of MNPs

All chemicals used, unless modified, were of analytical grade and obtained from Sigma Aldrich, South Africa. These included sodium hydroxide pellets (NaOH), ferrous sulphate heptahydrate (FeS0_4_·7H_2_O), oleic acid (surfactant), ferrous chloride hexahydrates (FeCl_3_·6H_2_O), titanium oxides, chitosan and ethanol (95%). The magnetised nanomaterials (Fe_2_O_4_-TiO_2_, Cu-Fe_2_O_4_, Fe_3_O_4_ and Chitosan-Fe_2_O_4_/TiO_2_) used in this study were engineered via co-precipitation techniques [8] and characterised at the DUT, Chemical Engineering Research Lab, Durban, South Africa. The MNP SEM/EDX micrographs revealed a high magnification of 10–50k× and landing energy capacity of 20 keV with a view size of 20.8 µm and width diameter of 4.5 mm to 6.5 mm.

#### 3.1.2. Inoculum and Wastewater Distribution

The inoculum and wastewater samples were collected from an anaerobic digester operated by a local South Africa municipal wastewater treatment facility in the KwaZulu–Natal province. Using the American Public Health Association (APHA) [27] protocol for wastewater characterisation, the assay of the feedstock was found to constitute pH (6.3 ± 2.6), chemical oxygen demand (3570 ± 78.6 mg COD/L), turbidity (200 ± 32.7 NTU), total solids (554 mg TS/L), volatile solids (419 mg VS/L) and color (1340 ± 55.4 Pt.Co).

### 3.2. Biochemical Methane Potential (BMP) Test

**Figure 6:**
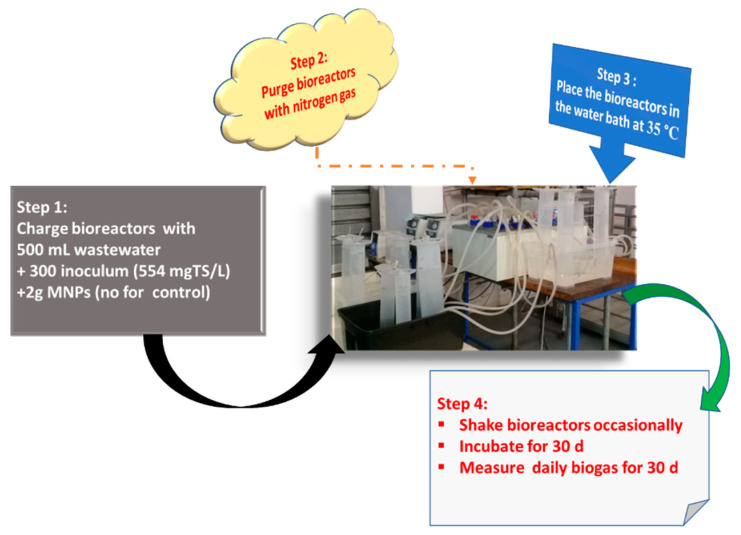
Schematic diagram of experimental procedure.

With a reactor volume of 1 L, working volume of 80% and 20% head space, five bioreactors (1 L Duran schott bottles) were experimentally set up as depicted in Figure 6. Each bioreactor was initiated with homogenised 0.5 L wastewater and 0.3 L inoculum and labelled A–E. Subsequently, bioreactors A–D were charged with 2 g of Fe_2_O_4_-TiO_2;_ Cu-Fe_2_O_4_; Fe_3_O_4_ and Chitosan-Fe_2_O_4_/TiO_2_ MNPs, respectively, whereas bioreactor E had no MNP additives. To enhance anaerobic conditions, each bioreactor was purged with nitrogen gas. In order to avoid thermal shocks in bioreactors and promote acclimatisation of the microbes, the system was left to stand for two days while adjusting the temperature from 27.5 °C to the mesophilic temperature of 40 °C. By using the downward displacement technique [4], the biogas produced was collected and monitored daily along with intermittent shaking of the bioreactors. After 30 days of incubation, each bioreactor supernatant sampled was analysed and estimated for the degree of efficiency of contaminant removal (4).

(4) $$\%\;degree\;efficiency=\frac{initial\;\left(feed\right)-final\;\left(after\;digestion\right)\;}{initial\;\left(feed\right)}\times 100$$

### 3.3. Digestate Analysis

**Figure 7:**
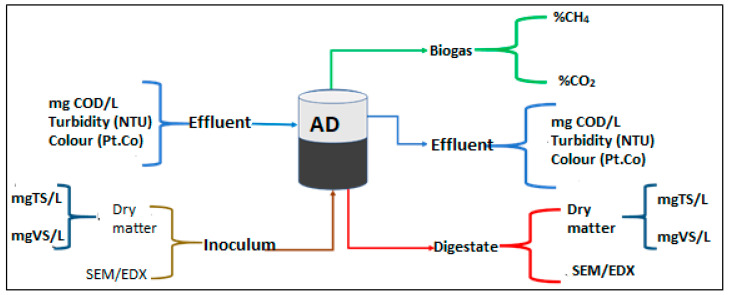
Schematic diagram of the component balance of the digestate input and output analysis.

The sludge was subjected to solids analysis before charging the bioreactors and after withdrawing the digestate from the bioreactors. Figure 7 represents the component balance of the influent and effluent water quality considered for characterisation using the standard protocols of analysing solids samples of APHA section 2540B [9]. Prior to knowing the mass of the crucibles used, they were first oven dried and allowed to cool in a desiccator. About 25 mL of the digestate was then weighed with the crucible and recorded as *Q*. At the temperature of 100 °C, the samples were then oven dried for 24 h. After drying, the sample was placed in a desiccator to cool, then immediately weighed with an analytical balance and recorded as *R*. The volatile solid content of the sample was determined using the furnace ignition at 550 °C for 1 h and the sample weighed and recorded as *S*. The estimated *TS* and *VS* were expressed with Equations (5) and (6), respectively:

(5) $$Total\;solids\;\left(TS\right)=\frac{Q-R\times 1000}{V_{S}}$$

(6) $$Volatile\;solids\;\left(VS\right)=\frac{R-S\times 1000}{V_{S}}$$

where *Q* is the mass (g) of crucible and sample after drying in oven, *R* is the mass of the crucible (g), *S* is the mass of the sample and crucible after calcination (g) and *V_S_* is the volume of the sample (mL).

### 3.4. Analytical Techniques

The pH was quantified with a Hannah pH–meter (HI98130, Hanna Instruments, Woonsocket, RI, USA). A HACH 2100N turbidity meter (Hach Company, Colorado, CO, USA) HACH DR 3900 within the wavelength of 455–635 nm (Hach Company, Colorado, CO, USA) was used for the COD and TKN measurements. The sludge samples were characterized using scanning electron microscopy and energy dispersive X-ray (SEM/EDX, FEI Nova NanoSEM 450 coupled with EDT and TLD detector) equipment based at the University of Cape Town, South Africa. The biogas composition analysis was carried out with a Geotech Biogas 5000 Portable Biogas Analyser (ISO17025) supplied by Keison Products, (Chelmsford Essex, UK).

## 4. Conclusions

In this study, the effects of magnetic nanoparticles (MNPs) on digestate for biogas production and decontamination were compared to the conventional treatment of municipality wastewater with no MNPs. This was carried out via biochemical methane potential (BMP) tests for 30 days using five bioreactors, A–D, charged with 2 g of Fe_2_O_4_-TiO_2_, Cu-Fe_2_O_4_, Fe_3_O_4_, and Chitosan-Fe_2_O_4_/TiO_2_ MNPs, respectively, while bioreactor E had no MNPs added. SEM/EDX results confirmed the presence of trace elements (MNPs) in the digestate matrix, with distinctive impacts and interactive features in the metabolic pathway via microbial activity. Addition of MNPs to the bioreactors was demonstrated to be efficient for the treatability of the wastewater, with over 75% of the COD, colour and turbidity removed. Comparatively, bioreactor A, which was charged with Fe_2_O_4_-TiO_2_ MNPs, showed itself as exceptionally viable in biostimulation of the AD process to increase biogas production (400 mL/day) and methane yield (100% CH_4_). Analysis of energy balance and cost were performed based on the influence of the MNPs in generating energy from biogas production. The addition of the MNPs to bioreactors A–D showed a considerable annual net energy profit gain of 170–320 ZAR/kWh, or 12–23 USD/kWh, as compared to the control bioreactor E of 68 ZAR/kWh, or 4.89 USD/kWh. Conclusively, bioreactor A with Fe_2_O_4_-TiO_2_ MNPs was estimated to have the highest energy profit (320.49 ZAR/kWh or 22.89 USD/kWh). Above all, the Fe_2_O_4_-TiO_2_ MNPs proved to be economically viable and had good potential to improve circular economy in anaerobically managed wastewater and waste management settings.

## Figures and Tables

**Table 1:** Decontamination efficiency of the bioreactors (A–E).

Parameter	Feed	A	B	C	D	E	Discharge Limit [10]
COD (mg/L)	3570 ± 79	181.5 ± 8	870 ± 12.5	354 ± 4.5	755 ± 5.6	1120 ± 7.6	75
COD removal (%)	-	94.92	75.63	90.08	78.85	68.63	-
Color (Pt.Co)	1340 ± 55	421 ± 25	628 ± 26	520 ± 17	450 ± 23	653.75 ± 23	<15
Color removal (%)	-	68.58	53.13	61.19	66.42	51.21	-
Turbidity (NTU)	200 ± 33	33.62 ± 27	58.35 ± 17	46.77 ± 5	39.77 ± 23	88.03 ± 23	<5
Turbidity removal (%)	-	83.19	70.83	76.61	80.12	55.99	-
VS/TS ratio	0.72	0.49	0.46	0.47	0.46	0.62	

**Table 2:** Cost estimation for the energy produced from 0.3 L sludge.

Item No	Item	Unit	A	B	C	D	E	[23]
	Type of sludge	Activated wastewater sludge						
1	Energy content of Methane	m^3^/h	0.0036	0.00198	0.00225	0.00294	0.00077	73.29
2	Energy produced (*E_A_*) (80% CH_4_ to electricity)	kW/h	0.00202	0.00113	0.00148	0.001283	0.00038	671
3	Energy (*E_H_*) used by the waterbath	kW/h	0.00058	0.00032	0.00036	0.00047	0.00012	601.6
4	Net energy $(E_{P})=E_{A}-E_{H}$	kW/h	0.0023	0.00127	0.00144	0.00188	0.00049	69.4
	Daily net energy cost estimated							
5	Energy cost (3.22 ZAR/kWh)	ZAR	0.00742	0.00409	0.00464	0.00606	0.00158	223.47
6	Energy cost (0.23 USD/kWh)	USD	0.00053	0.00029	0.00033	0.000432	0.00011	15.96
	Annual net energy cost estimated							
7	Energy cost (3.22 ZAR/kWh)	ZAR	320.49	176.67	200.31	261.57	68.43	
8	Energy cost (0.23 USD/kWh)	USD	22.89	12.62	14.30	18.68	4.89	

## Data Availability

Not applicable.

## Sample Availability

Not available.
